# Phonons and Thermal Expansion Behavior of NiSi and NiGe

**DOI:** 10.3389/fchem.2018.00331

**Published:** 2018-08-14

**Authors:** Prabhatasree Goel, Mayanak K. Gupta, Sanjay K. Mishra, Baltej Singh, Ranjan Mittal, Samrath L. Chaplot

**Affiliations:** ^1^Solid State Physics Division, Bhabha Atomic Research Centre, Mumbai, India; ^2^Homi Bhabha National Institute, Mumbai, India

**Keywords:** inelastic, neutron, phonons, ab-initio, negative thermal expansion, grunesien parameter

## Abstract

We have carried out first principles calculations of the vibrational and thermodynamic behavior in NiSi and isostructural compound NiGe. Phonon density of states has also been measured in NiSi using inelastic neutron scattering techniques. We find that the vibrational spectra of the two compounds are very different, due to the difference in the size and mass of Si and Ge. Interesting anomalous thermal behavior of NiSi due to anharmonic phonons is brought out well in our calculations, particularly the negative thermal expansion (NTE) along the b-axis of the orthorhombic unit cell. Large difference in thermal expansion behavior of NiSi and NiGe is very well reproduced by the calculations. Additionally, calculations enable to identify the phonon modes which lend major contribution to the negative thermal expansion behavior in NiSi, and reasons for negligible NTE in NiGe. Such typical representative modes at the zone-boundary along b-axis involve transverse vibrations of Si/Ge along c-axis.

**PACS numbers:** 78.70.Nx, 63.20.-e, 65.40.–b.

## Introduction

Transition metal silicides are considered important in microelectronics industry. These materials are used to reduce contact resistance at source, drain and gate terminals in standard complementary metal oxide semiconductor (CMOS) devices and very-large-scale integration (VLSI) technology. There are significant experimental and theoretical studies (Wu et al., 2005; Kim, 2015; Lin et al., 2015; Parshin et al., 2016; Stern and Madsen, 2016; Tang et al., 2017; Sidorova et al., 2018; Zhang et al., 2018a,b) on silicides to investigate their electronic and transport properties. Si-Ge alloys are the backbone of the metal-oxide semiconductor industry (Von Känel et al., 1992; Dahal et al., 2016; Lee et al., 2017; Simon et al., 2017; Kim et al., 2018). Lower resistivity, lower silicon consumption and higher stability make them better alternative to conventional silicides, like TiSi_2_ and CoSi_2_, for CMOS devices. Apart from its electronic industry applications, the compounds are reported to be good candidates for infrared sensor and ultraviolet photo detector applications. Detailed studies on the thermal behavior has been carried out on NiSiGe (Perrin et al., 2007) and FeNiSi (Simon et al., 2017) non-stoichiometric compositions, for fruitful integration of these derived compounds in the field of microelectronics.

NiSi (Franciosi et al., 1982; Lee et al., 2000; Detavernier et al., 2003; Jin et al., 2013), FeSi (Damascelli et al., 1997a,b), CoSi (Acun and Soyalp, 2012), and similar compounds (Kim, 2015) have been extensively studied with above applications in mind, and also for their various unusual physical and chemical properties (Weber et al., 2006; Lin et al., 2015; Geenen et al., 2018). NiSi (Connétable and Thomas, 2009) is structurally similar to NiGe. FeSi, a fascinating material, has been studied for its unusual magnetic and thermodynamic properties like thermal expansion, elastic behavior, heat capacity, Seebeck coefficients, magnetic susceptibility, insulator-metal transition with increasing temperature (Mandrus et al., 1995; Caracas and Wentzcovitch, 2004; Delaire et al., 2011; Parshin et al., 2016; Stern and Madsen, 2016). It is also described as a strongly correlated system, a possible Kondo insulator. Several electronic structure calculations have been reported on FeSi to understand its anomalous thermal expansion and to elucidate a link between its electronic density of states with the thermal disorder (Vočadlo et al., 2002). Phonon density of states have been studied in FeSi (Damascelli et al., 1997a,b; Racu et al., 2008), both experimentally and using *ab initio* molecular dynamics, to investigate softening of phonons (Delaire et al., 2013) and coupling between phonons and electronic structure in order to explain anomalous thermal expansion. Equation of state (Sarrao et al., 1994; Dobson et al., 2002) and elasticity of FeSi (Vočadlo et al., 2012) have been studied extensively to gain better understanding of the earth's core, which is mainly comprised of an alloy of iron and nickel, with a few light alloying elements like Si, C, S, H, and O.

Although NiSi and FeSi are not structurally similar at ambient conditions, their thermal expansion behavior is comparable. A cumulative study of the phonon density of states in NiSi using neutrons and first principles lattice dynamics calculations of thermal expansion would fill the gaps in complete understanding of these monosilicides and monogermanides. NiSi exhibits unusual thermal anisotropy (Donthu et al., 2004) with an expansion along the **a-** and **c-** direction while along **b-** direction there is significant contraction with increasing temperature. Although the overall thermal expansion coefficient has a positive value, yet the behavior along **b** direction makes it an interesting study. This behavior is consistent in both bulk and in thin films. In case of NiGe, there is a contraction along b- axis but it is not as large as in NiSi and FeSi. It would be very interesting to understand the differences in the behavior of NiSi, FeSi, and NiGe. The calculation of the elastic properties of NiSi and NiGe in comparison to the reported studies on FeSi would be useful to know the strength of these materials. High pressure theoretical studies on NiSi up to 500 GPa have already been reported (Lord et al., 2012; Vočadlo et al., 2012) for unraveling the mysteries surrounding the composition and behavior of earth's core. NiSi_x_Ge_(1−x)_ compounds are found to be promising in future microelectronics industry.

The crystal structure of NiSi and NiGe at ambient temperature is found to be orthorhombic (Pauling and Soldate, 1948), with space group Pnma. There are four Ni and four Si/Ge atoms in a unit cell. Although Ni is ferromagnetic, NiSi shows no magnetic ordering even up to lowest temperature around 0 K (Dahal et al., 2016). First principle studies to understand its high pressure behavior have been extensively reported (Jin et al., 2013). Anisotropy of thermal expansion in NiSi thin films have been studied (Perrin et al., 2007). Molar specific heat up to 400 K has been measured (Acker et al., 1999). Transport properties and neutron scattering to probe its magnetic ordering at 0.48 K have been reported. FeSi has a cubic P2_1_3 (B20) structure at ambient conditions. It transforms to the CsCl structure (Pm3m) at around 24 GPa and beyond 1950 K. NiSi adopts a B20 structure above 10 GPa and at around 900 K. It finally adopts a CsCl structure above 46 GPa and 1900 K.

This paper is a combined study of the vibrational properties of ambient phase of NiSi using neutron inelastic scattering experiments and theoretical calculations to understand the anomalous behavior of the thermal contraction along b-axis. First principles calculations have been carried out to derive the phonon density of states and thermal expansion behavior is obtained using *ab initio* lattice dynamics simulations. We also performed the calculation in NiGe. These studies will throw light on the specific phonon modes which are responsible for the anisotropic thermal behavior in NiSi, and the changes which come into play as we move from NiSi to NiGe. This is particularly interesting as the anisotropic thermal behavior is much less pronounced in NiGe.

## Experimental and computational details

Inelastic neutron scattering to study the phonon density of states in polycrystalline NiSi sample has been carried out using the Triple Axis Spectrometer at Dhruva reactor, Trombay. A sample of about 20 g of NiSi polycrystalline powder was used. All the measurements are carried out in the energy loss mode with constant momentum transfer (***Q***) geometry over a range of *Q*-values. The energy of the analyzer in various scans was kept fixed at 25 and 34.4 meV. In the incoherent one-phonon approximation (Carpenter and Price, 1985; Price and Skold, 1986), the measured scattering function *S*(*Q*,*E*), as observed in the neutron experiments, is related to the phonon density of states g^(n)^^(^E) as follows:

(1) $$\begin{array}{r} {\text{g}}^{\left(n\right)}\left(E\right)=A<\frac{{\text{e}}^{2W\left(Q\right)}}{{\text{Q}}^{2}}\frac{\text{E}}{n\left(E,T\right)+\frac{1}{2}\pm \frac{1}{2}}S\left(Q,E\right)> \end{array}$$

Where the + or – signs correspond to energy loss or gain of the neutrons respectively and $n\left(E,T\right)={\left[\exp\left(E/k_{B}T\right)-1\right]}^{-1}$. *A* and *B* are normalization constants.

The observed neutron-weighted phonon density of states is a sum of the partial components of the density of states due to the various atoms, weighted by their scattering length squares.

(2) $$\begin{array}{r} {\text{g}}^{\text{n}}\left(E\right)=\text{B}\underset{\text{k}}{\sum }\left\{\frac{4\text{π}{\text{b}}_{\text{k}}^{2}}{{\text{m}}_{\text{k}}}\right\}{\text{g}}_{{}^{\text{k}}}\left(E\right) \end{array}$$

Here *b*_*k*_, *m*_*k*_, and *g*_*k*_(*E*) are, respectively, the neutron scattering length, mass, and partial density of states of the *k*^th^ atom in the unit cell. The quantity between < > represents suitable average over all *Q* values at a given energy. 2*W*(*Q*) is the Debye-Waller factor averaged over all the atoms. The weighting factors $\frac{4\pi b_{k}^{2}}{m_{k}}$ for the various atoms in the units of barns/amu are: Ni: 0.331, and Si: 0.077.

The density functional theory calculations using VASP (Kresse and Furthmüller, 1996; Kresse and Joubert, 1999) along with PHONON software (Parlinksi, PHONON Software, 2003) is used to calculate the phonon frequencies of NiSi and NiGe in entire Brillouin zone. The software requires the forces on various atoms after displacement along various directions. These are obtained by computing the Hellman-Feynman forces on various atom in various configuration in a supercell with various (±x, ±y, ±z) atomic displacement patterns. The Hellman-Feynman forces on the atoms in the super cell have been calculated using density functional theory as implemented in the VASP software (Kresse and Furthmüller, 1996; Kresse and Joubert, 1999). NiSi and NiGe in orthorhombic phase (space group 62- Pnma) have 2 symmetrically inequivalent atoms in the unit cell, and total number of atoms in the primitive unit cell is 8. The force calculations is performed by displacing the symmetrically inequivalent atoms along (±x, ±y, ±z) directions. The energy cutoff is 1,000 eV and a 16 × 24 × 16 k point mess has been used to obtain energy convergence in total energy of the order of meV, which is sufficient to obtain the required accuracy in phonon energies. The Monkhorst Pack method is used for k point sampling (Monkhorst and Pack, 1976). The exchange-correlation contributions were included within PBE generalized gradient approximation (GGA) (Perdew et al., 1996). The structure is optimized with atomic pseudopotential with d^8^s^2^p^2^, s^2^p^2^ and s^2^p^2^ valence electrons in Ni, Si, and Ge atom respectively. The convergence criteria for the total energy and ionic forces were set to 10^−8^ eV and 10^−3^ eV Å^−1^, respectively.

## Results and discussion

### Phonon spectra

**Figure 1:**
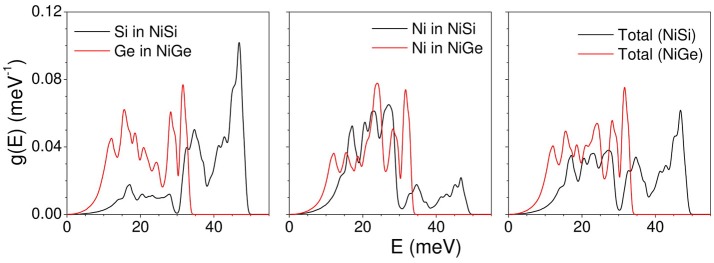
(Color online) The calculated total and partial density of states in NiSi (black line) and NiGe (red line).

The calculated total density of states in NiSi and NiGe along with respective partial contributions of various atoms is given in Figure 1. Replacing lighter Si in NiSi with Ge brings about a change in the lattice parameters and in the bond lengths. The lattice parameter as well as volume of NiGe is larger in comparison to that of NiSi. This is reflected in the phonon density of states in the two compounds. In case of NiSi, the phonon spectra extend to 50 meV, while for NiGe it extends only up to 34 meV. The spectral range of the partial contribution of Ni is different in both the compounds. Ge (72.63 amu) is heavier in comparison to Si (28.095 amu). The low energy peak in the partial density of states of Ge in NiGe is at 8 meV in comparison to that at 16 meV in Si spectrum of NiSi. The shift in the peak position may be mainly due to the mass difference of both the atoms in respective compounds. Further, Ni-Ge bond lengths are longer in comparison to that of Ni-Si and hence the phonon frequencies are lower. As a result, the span of the vibrational spectrum of NiGe is up to 34 meV is lower in comparison to that of 50 meV in NiSi.

**Figure 2:**
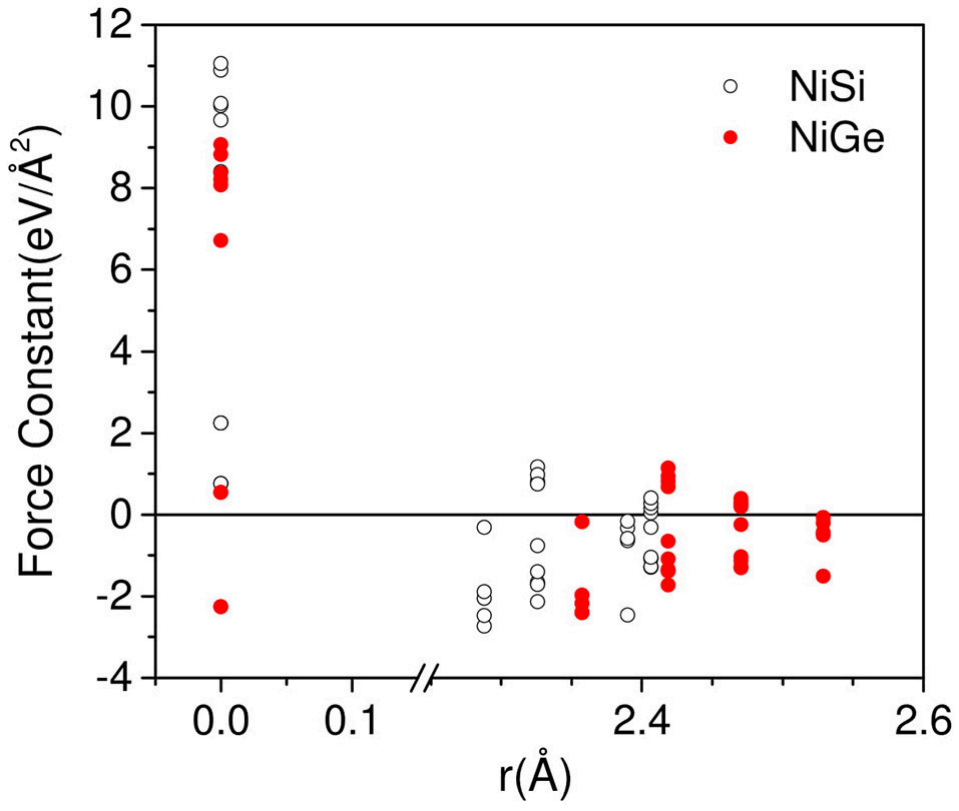
(Color online) The calculated force constant matrix components as a function of the interatomic distance in NiSi(open circle) and NiGe (solid circle).

In order to explain the difference in the range of phonon spectrum in NiSi and NiGe, we have plotted the force constant matrix components (Figure 2) as a function of the interatomic distance. Typically, the force constant Φ(*k, k*′) between a pair of atoms (k, k′) is determined from the force on the atom k' due to a small displacement of the atom k. On the other hand, the self force constant Φ(*k, k*) occurs due the force on the atom k when the same atom is displaced. The values at the zero distance (Figure 2) refer to the self force constants. The self force constants for NiGe are about 70% of the values for NiSi. The first nearest neighbor distances occur in the range of 2.2–2.6 Å in NiSi and NiGe, which correspond to Ni-Si and Ni-Ge bond lengths. We can see that the magnitude of force constant matrix elements in NiSi is larger than that in NiGe, which reflects in the phonon spectrum of these compounds. The phonon spectrum of NiSi extends up to 50 meV, while in NiGe the range is only up to 34 meV.

**Figure 3:**
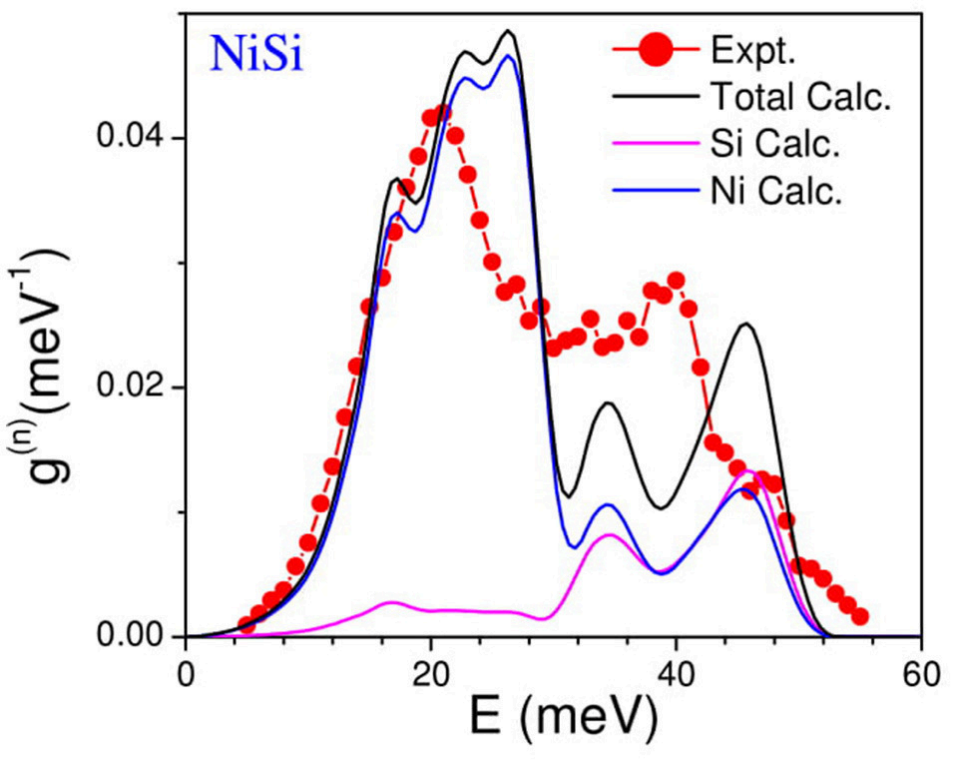
(Color online) Experimental and computed neutron weighted phonon density of states of NiSi. The calculated neutron cross-section weighted partial phonon density of states of various atoms is also shown. The calculated spectrum has been broadened by the energy resolution in the experiment.

The measured neutron weighted phonon density of states using inelastic neutron scattering in NiSi has been shown in Figure 3. The experimental spectra compare very well with the calculations. The phonon spectra extend up to about 50 meV. The calculated neutron cross-section weighted partial phonon density of states of various atoms (Figure 3) clearly shows that the first peak in the phonon spectrum at 20 meV has mainly contribution from the Ni atom, while above 30 meV both the Ni and Si atoms contribute equally. The general characteristics of the experimental features are very well reproduced by the calculations. We may note that the peak heights in the calculated spectrum do not match very well with the measured inelastic spectrum. This may be due to the known inherent limitations both in experiment (e.g., incoherent approximation) and theory.

**Figure 4:**
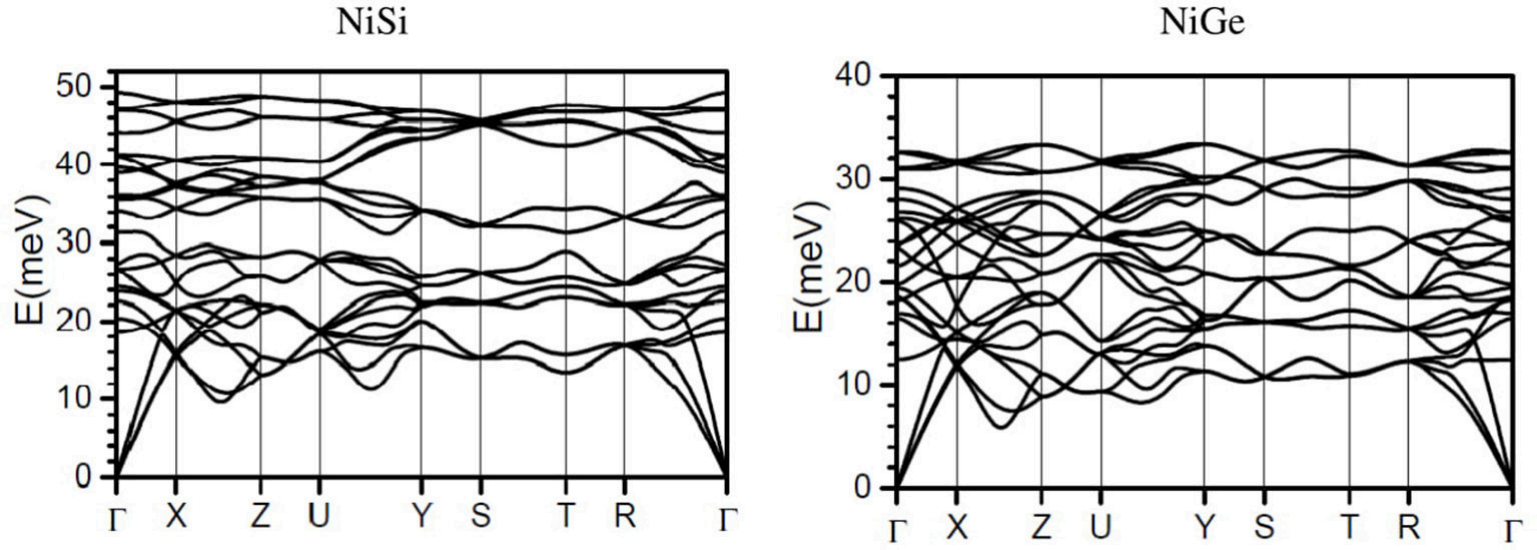
The calculated phonon dispersion curves in NiSi and NiGe along the high symmetry points in the Brillioun zone.

Phonon dispersion relation in NiSi and NiGe has been calculated (Figure 4). In accordance to the calculated phonon density of states, the spectral range of phonons in NiSi extends up to 50 meV, while the highest mode in NiGe is at around 34 meV. Comparison of the dispersion curve shows that the acoustic modes in NiSi extend up to 20 meV while the energy range in NiGe is up to 12 meV. These will give rise to difference in the elastic properties which arises from the slope of acoustic phonon branches along various high symmetry directions in the Brillion zone of both the compounds. The optic modes in NiSi are well separated and give rise to a three peak structure in the phonon spectrum (Figure 1), while in NiGe the modes are not separated and they give rise to a nearly broad peak (Figure 1).

The group theoretical decomposition of phonons at the zone center is given as:

$$\begin{array}{l} 4{\text{A}}_{\text{g}}+{2\text{B}}_{1\text{g}}+{4\text{B}}_{2\text{g}}+{2\text{B}}_{3\text{g}}+{2\text{A}}_{\text{u}}+{4\text{B}}_{1\text{u}}+2B_{2\text{u}}+{4\text{B}}_{3\text{u}} \end{array}$$

One each of the B _1u_, B_2u_, and B _3u_ modes are zero energy acoustic modes. The calculated Raman and infrared modes in NiSi and NiGe is given in Table 1. The comparison of zone center modes also shows that energies of various modes in NiSi extend up to about 50 meV while the maximum range in NiGe is up to 31 meV.

**Table 1:** The calculated Raman and infra red modes of NiSi and NiGe in meV units.

**Representation**	**NiSi**	**NiGe**
A_g_	24	17
	27	19
	36	23
	44	31
B_1g_	19	12
	41	29
B_2g_	27	18
	31	26
	49	33
	41	27
B_3g_	24	19
B_3g_	39	24
A_u_	20	18
	36	22
B_1u_	27	16
	34	26
	47	33
B_2u_	40	28
B_3u_	22	20
	36	23
	47	31

The calculated elastic constants for both the compounds are given in Table 2. It can be seen that all the elastic constants in NiSi have larger values in comparison to that in NiGe. This compares very well with the observed slopes of the dispersion curves in both the compounds. Similarly the bulk modulus value of NiSi (162.6 GPa) is found to be larger in comparison to that of NiGe (119.5 GPa). The bulk modulus of NiSi has been measured using synchrotron-based X-ray powder diffraction method (Lord et al., 2012) and found to be 165 ± 3 GPa, which is in very good agreement with our calculation.

**Table 2:** The calculated elastic constants of NiSi and NiGe in GPa units.

**Elastic Constants**	**NiSi**	**NiGe**
*C_11_*	265	194
*C_12_*	160	121
*C_13_*	87	84
*C_22_*	215	156
*C_23_*	143	91
*C_33_*	227	157
*C_44_*	108	102
*C_55_*	131	71
*C_66_*	127	104
*B*	163	119

### Thermal expansion behavior

Both NiSi and NiGe are reported to show negative thermal expansion along the **b**-direction, while expansion coefficient is positive along **a-** and **c**-axis. The overall volume thermal expansion coefficient is positive. The anisotropic thermal expansion behavior can be computed under the quasiharmonic approximation. Each phonon mode of energy *E*_*qj*_ (*j*^*th*^ phonon mode at point q in the Brillouin zone) contributes to the thermal expansion coefficient, which is given by following relations for an orthorhombic (Mittal et al., 2018) system:

(3) $$\begin{array}{r} \alpha_{a}\left(T\right)=\frac{1}{V_{0}}\underset{q,j}{\sum }C_{v}\left(q,j,T\right)\left[{s_{11}\Gamma_{a}+s_{12}\Gamma_{b}+s}_{13}\Gamma_{c}\right] \end{array}$$

(4) $$\begin{array}{r} \alpha_{b}\left(T\right)=\frac{1}{V_{0}}\underset{q,j}{\sum }C_{v}\left(q,j,T\right)\left[{s_{21}\Gamma_{a}+s_{22}\Gamma_{b}+s}_{23}\Gamma_{c}\right] \end{array}$$

(5) $$\begin{array}{r} \alpha_{c}\left(T\right)=\frac{1}{V_{0}}\underset{q,j}{\sum }C_{v}\left(q,j,T\right)\left[s_{31}\Gamma_{a}+s_{32}\Gamma_{b}+s_{33}\Gamma_{c}\right] \end{array}$$

Where V_0_ is the unit cell volume, and *s*_*ij*_ are elements of elastic compliances matrix *s* = *C*^−1^. *C*_*v*_ *(q, j, T)* is the specific-heat contribution from the phonon of energy *E*_*q, j*_. Γ_*a*_, Γ_*b*_ and Γ_*c*_ are the anisotropic mode Grüneisen parameters of phonon of energy *E*_*q, j*_ as given by (Grüneisen and and Goens, 1924),

(6) $$\begin{array}{r} \Gamma_{l}\left(E_{q,j}\right)=-{\left(\frac{\partial lnE_{q,j}}{\partial lnl}\right)}_{l^{\prime }};\;l,\;l^{\prime }=a,b,\;c \end{array}$$

The volume thermal expansion coefficient for a orthorhombic system is given by:

$$\begin{array}{l} \alpha_{V}=\left(\alpha_{a}+{\alpha_{b}+\alpha }_{c}\right) \end{array}$$

**Figure 5:**
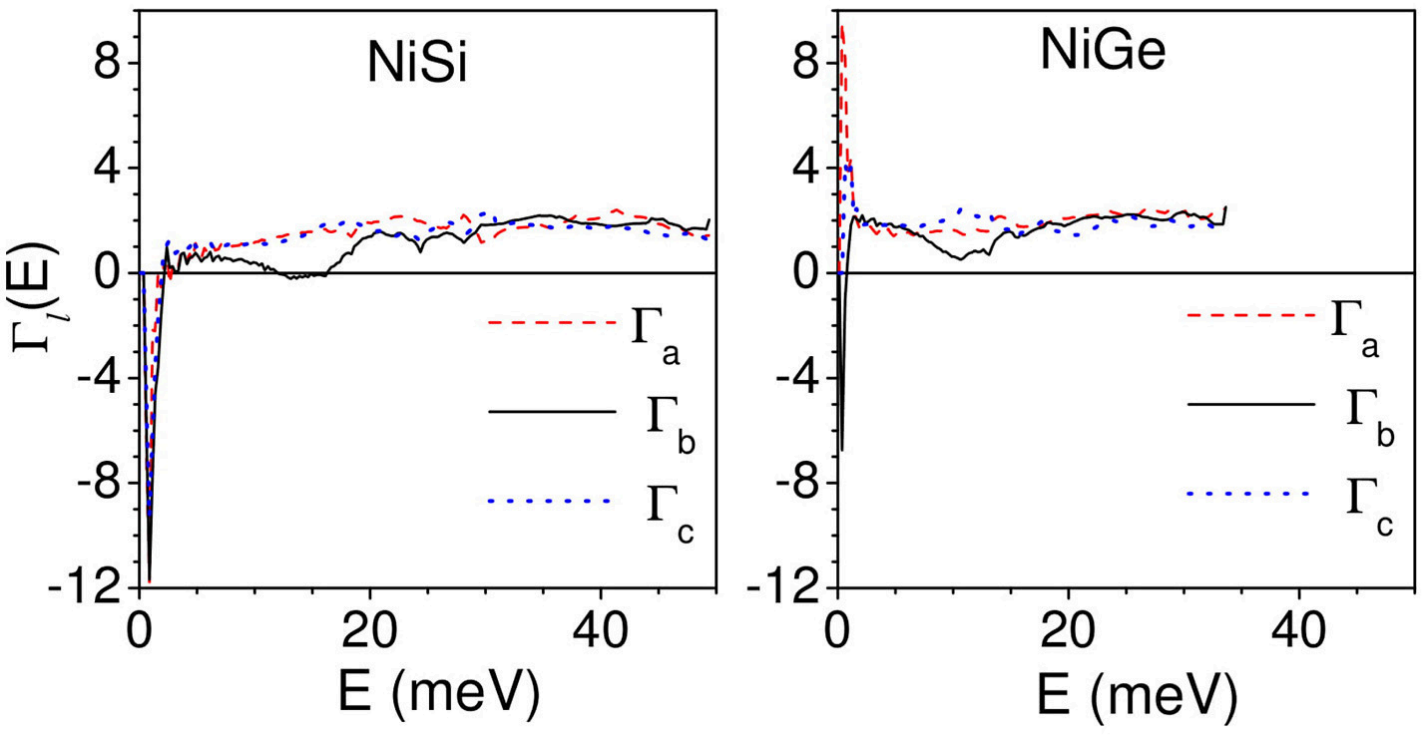
(Color online) Calculated variation of anisotropic Grüneisen parameters with energy averaged over all the phonon modes in the Brillouin zone in NiSi and NiGe along a, b, and c directions.

The calculation of anisotropic thermal expansion requires calculation of anisotropic Grüneisen parameters. The calculations of Γ_*l*_(*E*) are performed by applying an anisotropic stress by changing the lattice constant “a” and keeping the “b” and “c” parameters constant; and vice versa following eq.(6). Figure 5 gives the calculated variation of anisotropic Grüneisen parameters with energy averaged over all the phonon modes in the Brillouin zone in NiSi and NiGe along a, b, and c directions.

As shown in Figure 5, for NiSi, the acoustic modes up to about 2.5 meV have negative values of Γ_*l*_(*E*) (*l* = *a, b.c*) of about −12, while in NiGe the Γ_*l*_(*E*) have relatively smaller negative values of −6 along b- and c- axis and positive values of +8 along a- axis. Further for NiSi, the modes in the energy range of 12–16 meV show small negative values of −0.2 along b-axis. The Γ_*l*_(*E*) (*l* = *a, b.c*) in the remaining part of the phonon spectrum have positive values. In case of NiGe, the Γ_b_ shows a dip in the energy range of 12–16 meV with positive values of +0.5. Above 2.5 meV, Γ_*l*_(*E*) in NiGe along the three axes have positive values.

**Figure 6:**
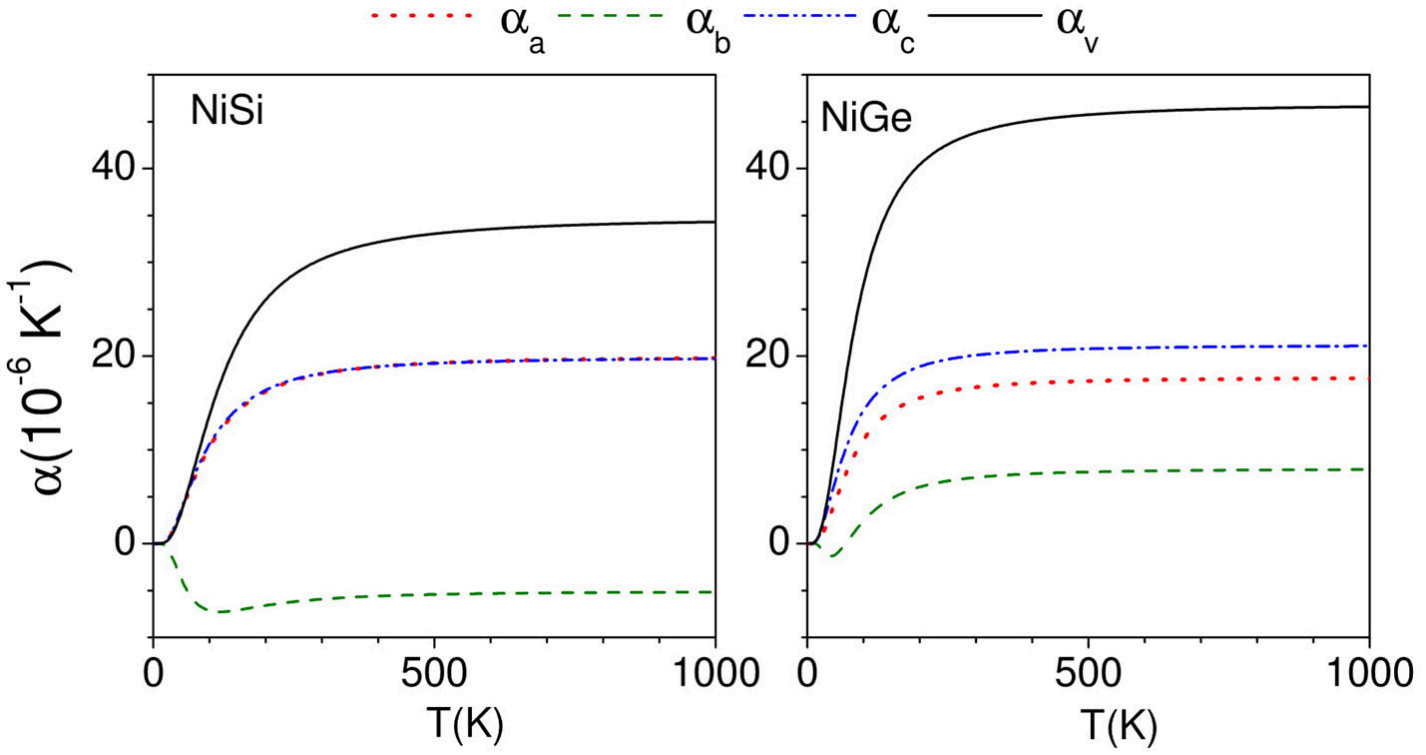
(Color online) Calculated anisotropic behavior of the linear thermal expansion coefficients along a, b, and c directions as a function of temperature in NiSi and NiGe.

The calculated thermal expansion coefficients as obtained from equations (3)–(5) are shown in Figure 6. We find that for NiSi, the thermal expansion coefficients are calculated to be positive along a and c-axes, while it is negative along b-axis. Similarly for NiGe, the α_b_ has small negative values up to about 100 K and it is positive above 100 K. The α_*l*_ (*l* = a, c) along a and c-axis are found to be positive in the whole temperature range. The experimental thermal expansion coefficient α_b_ has large negative value of −16.1 × 10^−6^ K^−1^ for NiSi, while in NiGe α_*b*_ has small negative values of ~ −0.4 × 10^−6^ K^−1^. Our calculations are not able to quantitatively reproduce the negative values along b-axis in both the compounds, while qualitatively they agree very well with the experimental observations of a large difference in α_*b*_ in both the compounds.

**Figure 7:**
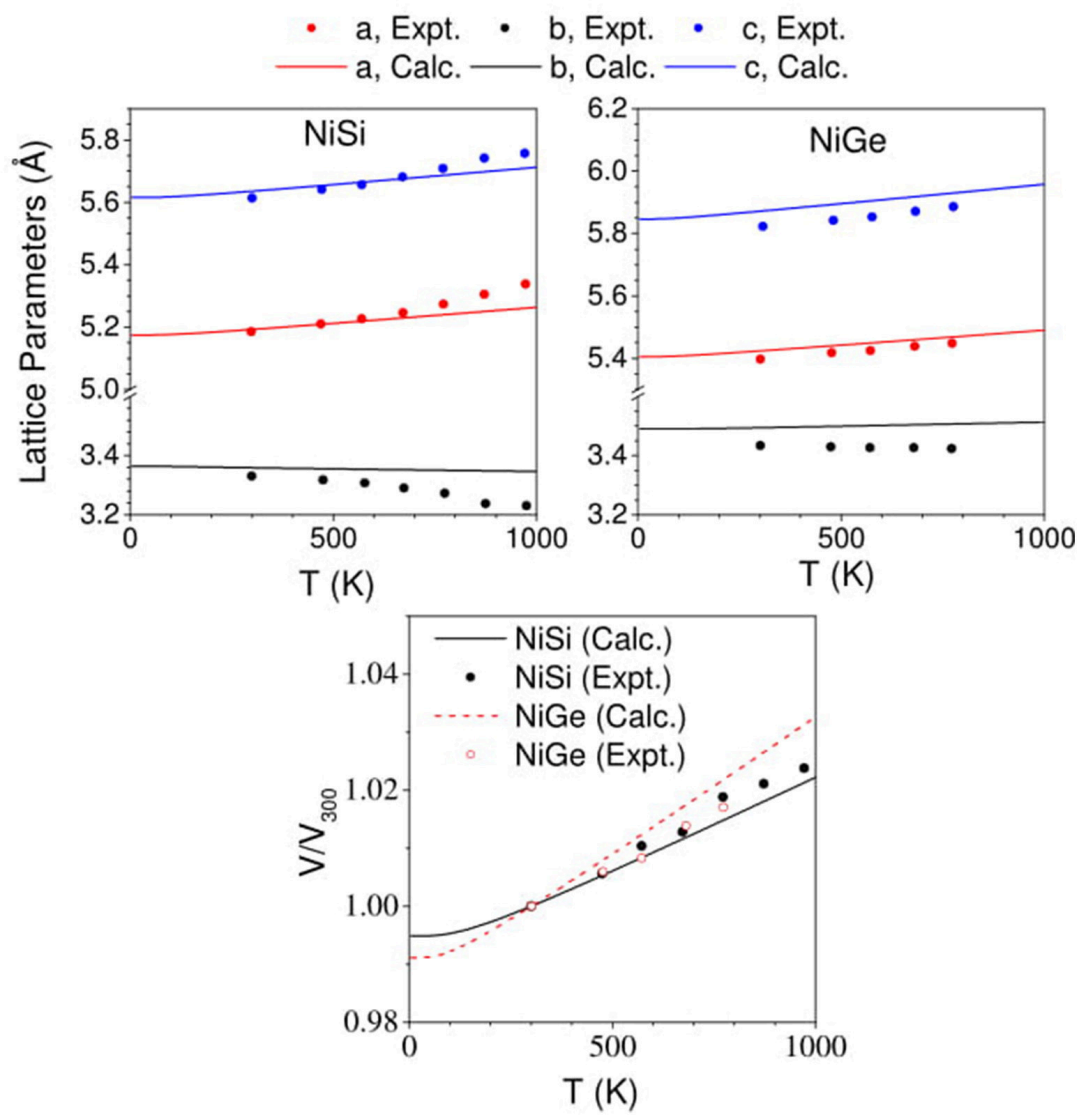
(Color online) Change in lattice parameters and volume of NiSi and NiGe with increasing temperature. Our computed values (solid lines) in comparison with experimental data (Perrin et al., 2007) (solid symbols).

The comparison between the experimental (Perrin et al., 2007) and calculated change in the lattice parameters with increasing temperature is given in Figure 7, along with change in unit cell volume of the two compounds. The change in a- and c- lattice parameters as computed in this work is in good agreement with reported experimental data (Perrin et al., 2007). However, along the b- axis the contraction with increasing temperature is found to be underestimated in our calculations than from the experiments (Perrin et al., 2007). The change in b- lattice parameter is found to be more abrupt in the experimental observations for NiSi than that in our calculated values. The calculations are found to underestimate the contraction along the b-axis in NiSi, while for NiGe the comparison between the experiments and calculations seems to show good agreement with the reported data. Further, we find that the change in the volume of NiSi (Figure 7) from our calculations is found to be underestimated in comparison to the experimental data. The calculated volume is found to be slightly overestimated in NiGe (Figure 7).

**Figure 8:**
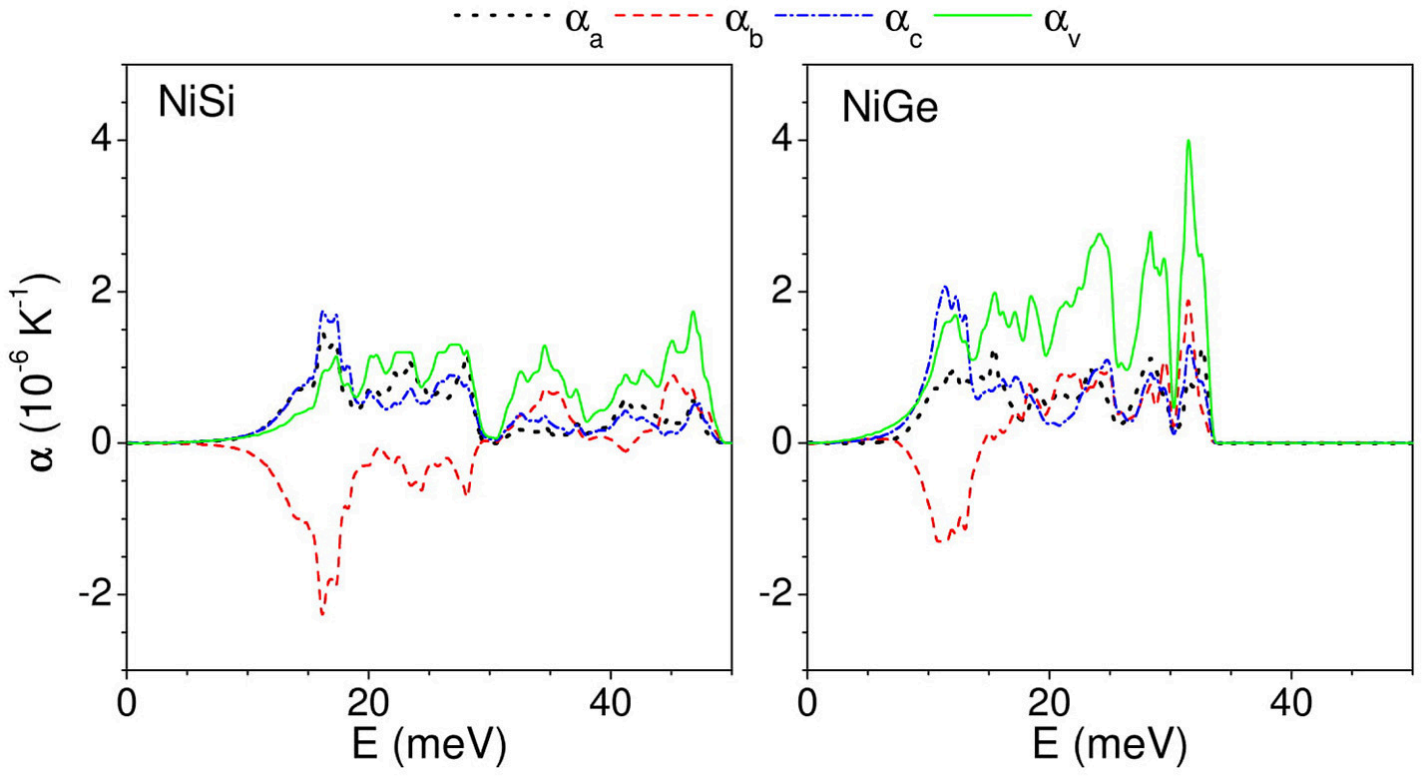
(Color online) The contribution to anisotropic linear and volume thermal expansion coefficients at 300 K, from phonon mode of energy E averaged over the Brillouin zone.

As described above, the thermal expansion coefficient at a given temperature is an outcome of contribution of phonon modes of different energies in entire Brillouin zone. To get a better insight, we have calculated (Figure 8) the contribution of phonons of energy E, averaged over Brillouin zone, to the linear thermal expansion coefficient at 300 K. In case of NiSi, phonons from entire Brillouin zone contribute to positive expansion along both a- and c- axis. Interestingly, phonons below 30 meV contribute to negative expansion coefficient along b- axis. We find that phonon modes below 30 meV contribute in different ways along the a-, c-, and b- axis. Further, in case of NiGe, we find similar behavior of phonon contribution along a- and c- axis i.e., positive contribution to thermal expansion coefficient. However, along the b- axis, phonons up to 15 meV contribute to negative expansion coefficient. We find that the range of phonon energies contributing to NTE along b- axis is significantly different for NiSi and NiGe; and this leads to significant difference in the magnitude of negative thermal expansion along b- axis in these compounds. The important inference of the above analysis is that the modes below 15 meV strongly contribute to NTE along b- axis in both the compounds.

As mentioned in the computation details above, the thermal expansion coefficient along different axes is given by the sum of the products of anisotropic Γ and the elastic compliance components relevant to the given direction. These values have been calculated and given in Table 3. For NiSi and NiGe, thermal expansion α_b_(T) is given by including the values of the individual compliance values from Table 3:

$$\begin{array}{l} For\;NiSi\;\alpha_{b}(T)=\frac{1}{V_{0}}\sum_{q,j}^{}C_{v}(q,j,T)[-0.059\Gamma_{a}+0.129\Gamma_{b}\\ \;+\;0.059\Gamma_{c}] \end{array}$$

$$\begin{array}{l} For\;NiGe\alpha_{b}(T)=\frac{1}{V_{0}}\sum_{q,j}^{}C_{v}(q,j,T)[-0.072\Gamma_{a}+0.146\Gamma_{b}\\ \;-0.046\Gamma_{c}] \end{array}$$

In case of NiSi, the values of Γ_a_ and Γ_c_ are comparable for both the compounds, and they cancel out: so only 0.129Γ_b_ term contributes. As Γ_b_ values are negative, the net value of α_b_ becomes negative. In case of NiGe, sum of the first and the third term is less than the second term, and since Γ_b_ values are positive, the net value of α_b_ is positive. The Γ_b_ values for different modes in the range of 12–18 meV (NiSi) are almost zero or negligibly small negative, while in case of NiGe they are small positive. As a result, the value of the thermal expansion coefficient in case of NiSi becomes more negative as compared to NiGe. Hence negative thermal expansion is more pronounced in NiSi.

**Table 3:** The calculated elastic compliance of NiSi and NiGe in (10^−3^ GPa^−1^) units.

**Elastic Constants**	**NiSi**	**NiGe**
*S_11_*	70	103
*S_12_*	−59	−72
*S_13_*	10	−14
*S_22_*	129	146
*S_23_*	59	−46
*S_33_*	77	98
*S_44_*	93	98
*S_55_*	77	142
*S_66_*	78	96

**Figure 9:**
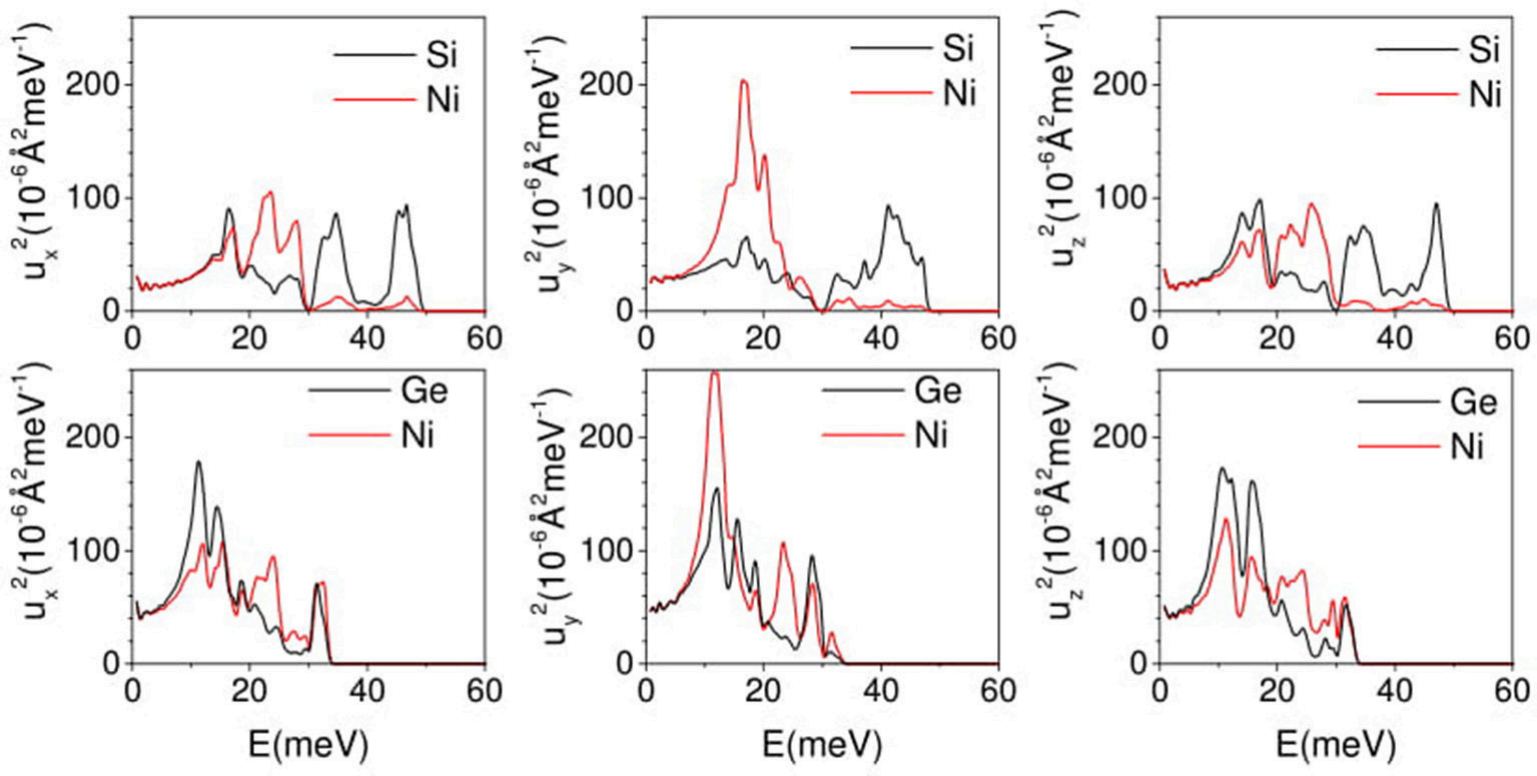
(Color online) The calculated anisotropic mean square displacement of various atom in NiSi and NiGe as a function of phonon energy E averaged over Brillouin zone at 300 K.

In case of NiSi, the specific modes exhibiting negative values in α_b_ and the vibrational amplitude of the atoms in these low energy modes would throw light on the physical mechanism involved in NTE. This in turn would eventually lead us to better understanding of the observed negative thermal expansion along b-direction in NiSi. The substitution of Ge in place of Si, brings about changes in the lattice which cancels out the possibility of similar behavior in NiGe. We have calculated the mean square amplitudes of various atom in NiSi and NiGe along a-, b-, and c- axis as a function of phonon energy to understand the mechanism of NTE along b- axis in both the compound (Figure 9). In Figure 8, we have observed that the NTE along b- axis in NiSi (NiGe) is mainly contributed by phonon modes of energy 8–20 meV. However, along a- and c- axis all the phonon modes contribute to positive expansion behavior (Figure 8). The density of states around 8–20 meV energy is very large, hence these modes play the dominating role in thermal expansion behavior of NiSi(NiGe). In Figure 9, the mean square displacements of Ni and Si below 20 meV show similar amplitude along a- and c- axis. However, along b- axis the magnitude of Ni is about 4 times than that of Si. In NiGe compound, the mean square displacements of Ge along b-axis are large in comparison to that of Si in NiSi. The ratio of u^2^ values, along b-axis, for Ni to Ge is only about 1.5. These results of mean-square amplitudes clearly bring out the similarity between the dynamics along the **a**- and **c**- axes and a different behavior along the b-axis, particularly for the modes in the 8–20 meV range. This dynamical behavior is also reflected in the nature of the anisotropic thermal expansion behavior along the b-axis.

**Figure 10:**
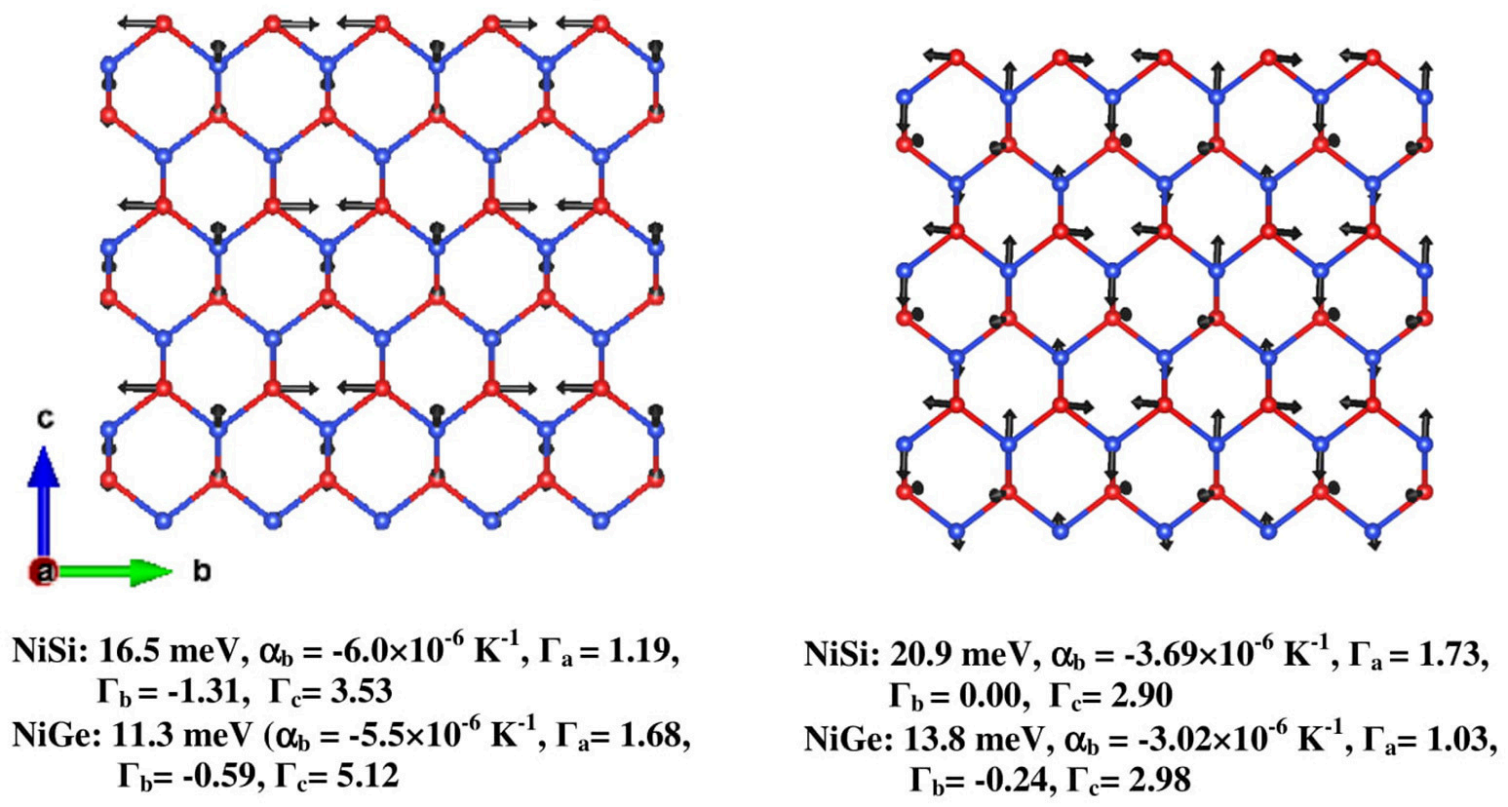
(Color online) The displacement pattern of two representative modes at the Brillouin zone boundary (0 1/2 0) responsible for negative thermal expansion in NiSi and NiGe. The numbers below the figures give the phonon energy, α_b_ at 300 K, Γ_a_, Γ_b_, and Γ_c_, respectively. Key: Ni–Red and Si/Ge–Blue.

The NiSi structure consists of hexagonal sheets formed by Ni and Si atoms stacked along the **a**- axis. In Figure 10, we have given two of such representative modes at Y(0 1/2 0) point of 16.5 and 20.9 meV, which lead to negative expansion along **b**- axis. From Eq. (4), the 16.5/20.9 meV mode gives significant contraction along b- axis (α_b_ = −6.0 × 10^−6^ K^−1^/−3.69 × 10^−6^ K^−1^, Γ_a_ = 1.19/1.73, Γ_b_ = −1.31/0.00, Γ_c_ = 3.53/2.90) and expansion in the a–c plane. The same representative modes in NiGe appear at an energy of 11.3/13.8 meV and contribute to NTE along b- axis (α_b_ = −5.5/−3.02 × 10^−6^ K^−1^, Γ_a_ = 1.68/1.03, Γ_b_ = −0.59/−0.24, Γ_c_ = 5.12/2.98). The displacement pattern of 16.5 meV mode shows that, two of the three neighboring Ni atoms in a given hexagon move anti parallel to each other along b- axis, while the third Ni is almost at rest; and similarly two of the Si atoms vibrate along the c-axis. In case of 20.9 meV mode, all the three Ni atoms and the Si atoms in the hexagon have significant displacements. Both of these modes propagate along the b-axis, and involve transverse Si vibrations largely along the c-axis. It may be noted that transverse vibrations may contribute to NTE since it causes a negative stress in the longitudinal direction.

The displacement patterns of these modes at 16.5/20.9 meV in NiSi are very similar to the modes at 11.3/13.8 meV for NiGe. However, in NiGe, other higher energy modes which give contribution to positive expansion are well below 30 meV and significantly contribute at ambient temperature, thus nullifying the NTE along b- axis from the above phonon mode, while in NiSi the high-energy modes are distributed up to 50 meV, hence positive contributions from these modes are less significant.

## Conclusions

Inelastic neutron scattering study of the phonon density of states in NiSi is reported. The measured data are in very good agreement with our calculations. We also report calculations on isostructural compound NiGe. Due to the heavier and bigger Ge atom, the phonon density of states of the two compounds differs in their total span. The contribution of Ni to the phonon density of states in both the compounds differs. NiSi shows large NTE along b-axis, while NiGe compound has very small NTE behavior. The difference in the negative thermal expansion along b-axis in both compounds is brought out very well in our calculations. The different thermal expansion along the b-axis for the two compounds could be understood in terms of the respective values of the Grüneisen parameters as well as the elastic compliances. We have identified representative phonon modes involving transverse vibration of Si/Ge atoms, which contribute significantly to the phenomenon of NTE along b-axis.

## Author contributions

PG contributed in inelastic neutron scattering experiment, analysis and interpretation of experiment and ab-initio calculation, and writing of the manuscript. MG and BS contributed in inelastic neutron scattering experiment, ab-initio calculations, interpretation of experiment and ab-initio calculation, and writing of the manuscript. SM contributed in inelastic neutron scattering experiment, analysis and interpretation of experiment and ab-initio calculation and writing of the manuscript. RM formulated the problem, contributed in inelastic neutron scattering experiment, interpretation of experiment and ab-initio calculation, and writing of the manuscript. SC formulated the problem, contributed in interpretation of experiment and ab-initio calculation, and writing of the manuscript.

### Conflict of interest statement

The authors declare that the research was conducted in the absence of any commercial or financial relationships that could be construed as a potential conflict of interest. The reviewer LW and handling Editor declared their shared affiliation.
